# Serum albumin, cognitive function, motor impairment, and survival prognosis in Parkinson disease

**DOI:** 10.1097/MD.0000000000030324

**Published:** 2022-09-16

**Authors:** Shujun Sun, Yiyong Wen, Yandeng Li

**Affiliations:** a Department of Neurology, The Frist People’s Hospital of Changde City, Changde, Hunan 415003, China; b Department of General Practice, The Frist People’s Hospital of Changde City, Changde, Hunan 415003, China.

**Keywords:** cognitive function, human serum albumin, motor impairment, Parkinson disease, prognosis

## Abstract

The role of albumin in Parkinson disease (PD) is not well understood, our study will investigate the association between the serum albumin level and risk of dementia, motor impairment, as well as survival outcome in PD. Data were obtained from the publicly available dataset in the DRYAD database (https://datadryad.org/). The original prospective study enrolled patients with PD from a single center in Japan between March 2004 and November 2007. Due to missing values, 242 and 274 participants were included in the study, in which we aimed to, respectively, analyze the relationship between serum albumin and cognitive function as well as motor impairment; additionally, 264 participants were included to assess the association between baseline serum albumin levels and risk of PD-related death with a median follow-up of 5.24 years. Compared to patients of the low tertile of albumin levels, Mini-Mental State Examination (MMSE) of patients of middle tertile increased 2.09 [95% confidence interval (CI) (0.45, 3.73), *P* = .013], independent of age, sex, PD duration, modified Hoehn-Yahr (mHY) stage, C-reactive protein (CRP) level, and use of nonsteroidal anti-inflammatory drugs (NSAIDs). Further analysis revealed a positive curvilinear association between albumin and MMSE, with cutoff values of 3.9. As concentration serum albumin increased, the risk of severe motor impairment was grown [odds ratio (OR) 0.34 (95% CI 0.14,0.8), *P* = .013] after adjustment by age, sex, PD duration, MMSE scores, CRP level, and use of NSAIDs. Albumin levels increased per unit of mg/dL, and the risk of PD-related death reduced 0.74-fold with 95% CI (0.15, 0.86) (*P* = .021), independent of age, sex, PD disease duration, mHY stage, CRP levels, use of NSAIDs, and MMSE. Higher serum albumin levels were significantly association with the better cognitive function when albumin was <3.9 mg/dL, and played a protective role in severe motor impairment and PD-related death.

## 1. Introduction

Parkinson disease (PD) is the second most common neurodegenerative disorder, in which the main clinical manifestations include bradykinesia, rigidity, postural instability, and tremor. PD has an estimated prevalence of 0.3% in the United States and a similar prevalence in Europe.^[1,2]^ Population aging contributes to the fast-growing prevalence of PD; indeed, by the 2030s, it is estimated that Asia will have over 60% of the world’s population aged ≥65 years. Thus, it appears that the majority of patients with PD worldwide will be from the Western Pacific Region.^[3]^

Albumin is the most abundant plasma protein in the body, as well as a major component of cerebrospinal fluid, and it has several biochemical properties, including antioxidant and anti-inflammatory properties. Albumin also has neuroprotective effects, which are partly attributed to modulation of intracellular signaling of neuronal or glial cells and antioxidant properties.^[4]^ In Alzheimer disease (AD), albumin can suppress amyloid formation and block further accumulation of peptide amyloid beta (Aβ) protein; also, the serum albumin level was inversely associated with Aβ deposition and Aβ positivity.^[5]^ In oldest-old patients with AD, dementia severity was found to be significantly associated with serum albumin levels.^[6]^ In a multicenter, randomized, controlled clinical trial, patients with AD treated with plasma exchange with 5% albumin showed improvement in memory and language functions.^[7]^ In conclusion, albumin may have a protective effect on AD and has a similar effect in PD.

It is well known that PD is characterized pathologically by the presence of Lewy bodies and dopaminergic neuronal death. The misfolding and uncontrolled aggregation of alpha synuclein (αS), an important component of Lewy bodies, is linked to the onset and progression of PD.^[8]^ Human serum albumin (HSA) slows the aggregation of αS significantly.^[9]^ Also, the toxicity of aS oligomers can be inhibited by HSA.^[10]^ In this context, it is reasonable to assume that baseline albumin levels may be related to the prognosis of PD. It is well known that one of the main symptoms of PD is dyskinesia, and one of the most common complications of PD is the development of dementia and cognitive dysfunction, which may present in varying degrees. However, it is unclear whether serum albumin is related to cognitive function, motor impairment, and survival outcome. Most previous studies have focused on the functional prognosis of PD; however, the survival prognosis is also an important endpoint; therefore, we examined the relationship between serum albumin motor impairment, and survival outcome in our study. The available data from a retrospective study conducted in Japan (https://datadryad.org/) were reanalyzed in order to evaluate the role of albumin in PD.

## 2. Materials and Methods

### 2.1. Study design and participants

Our study consists of a secondary analysis of a single-center retrospective study that was conducted at the Department of Neurology, National Regional Center for Neurological Disorders, and National Hospital of Utano. Patients with PD treated at the Department of Neurology between March 2004 and November 2007 were enrolled in this study. PD diagnosis was based on the United Kingdom Parkinson’s Disease Brain Bank Diagnostic Criteria, and enrollment criteria were identical to that of the original study.^[11]^ Open access data were extracted from the DRYAD database (https://datadryad.org/). The raw data can be freely obtained from the following website, and a secondary analysis was conducted using rational citation (Dryad data package: Sawada, Hideyuki et al,^[11]^ Data from: Baseline C-reactive protein level and life prognosis in PD, Dryad, Dataset, https://doi.org/10.5061/dryad.63vc5). This secondary analysis was undertaken at the Department of Neurology, Changde First People’s Hospital, Changde, Hunan Province, China.

A total of 313 patients were included in the original study; however, in this secondary analysis, data of patients missing serum albumin values and those that died from causes unrelated to PD were eliminated to clarify the association between baseline albumin levels and prognosis. Similarly, data of patients with missing Mini-Mental State Examination (MMSE) scores were removed to evaluate the role of albumin in cognitive function. The median follow-up time was 1912 days (5.24 years).

The original study was approved by the Bioethics Committee of the National Hospital of Utano (approval no. 26–4). Due to the retrospective nature of the study and anonymity of the collected data, the Bioethics committee waived the need for informed consent.^[11]^

### 2.2. Data collection and measurements

The database file was downloaded from DRYAD, and data for the following variables were obtained: age, sex, serum albumin, C-reactive protein (CRP) concentrations, PD disease duration, modified Hoehn-Yahr (mHY) stage, nonsteroidal anti-inflammatory drugs (NSAIDs), and MMSE, all above information were collected on the date of patient recruitment. The mHY stage reflected the severity of motor impairment of PD and was evaluated in the “ON period” in patients with motor fluctuations, while the MMSE scores and CRP values represented the cognitive function and inflammatory state, respectively. In the original study, data on the use of NSAIDs were also collected, since ibuprofen has been reported to be associated with PD risk reduction.^[12]^ In survival time analysis, the time from study enrollment to the endpoint was presented as the observation period, and the endpoint was defined as the date of death or May 16, 2014. Variables other than survival status and follow-up time were collected upon study enrollment.^[11]^

PD-related death was defined as an endpoint in the secondary analysis. Pneumonia, suffocation, dehydration, fracture due to falls, and sudden death were considered PD-related deaths, since these events are often seen in PD and are typically associated with death. In contrast, vascular deaths or cancer-related deaths were not considered to be associated with PD.^[11]^

### 2.3. Statistical analysis

Data are presented as mean ± standard deviation (SD) or median (interquartile range) based on data distribution for continuous variables, and as percentages and frequencies for categorical variables. We retrieve missing covariate data with statistical estimates of missing values. The median was used as an imputation value for continuous variables, and 9999 was used as an imputation value for missing covariate of categorical variable. This allowed researchers to utilize the collected data in an incomplete dataset. Differences among groups (albumin tertiles) were detected with one-way analysis of variance (ANOVA) for continuous variables and chi-square test for categorical variables. Linear regression analyses were used and partial regression coefficients (Bs) and 95% confidence intervals (CIs) were calculated for cognitive function with serum albumin levels. Severe motor impairment was defined as the mHY stage more than or equal to 4 in this study. A potential relationship of serum albumin with the risk of PD-related death was also evaluated using Cox regression analyses and hazard ratios (HRs) and 95% CIs were calculated. Both nonadjusted and multivariate-adjusted models were used. In our study, the Cox regression models were adjusted for all variables using logistic regression and MMSE. Linear regressions were used to conduct trend tests, with the median values of each albumin tertile used as continuous variables in the models. We also assessed the nonlinear relationship using the generalized additive model, and if a nonlinear relationship existed, the threshold effect was further determined using a two-piecewise linear regression model based on the smoothing curve. Subgroup analyses of the association between serum albumin levels and cognitive function were conducted with stratified Cox regression models based on age, sex, PD disease duration, mHY stage, and use of NSAIDs. Likelihood ratio tests were used to test the interaction across subgroups. Survival curves of patients with PD were generated and compared with baseline albumin tertiles. All analyses were performed using the statistical packages R version 3.3.2 (http://www.R-project.org, The R Foundation) and Free Statistics software version 1.1. All *P* values were two-tailed, and a *P* value <.05 was considered statistically significant.

## 3. Results

### 3.1. Baseline characteristics of study participants

**Figure 1.**
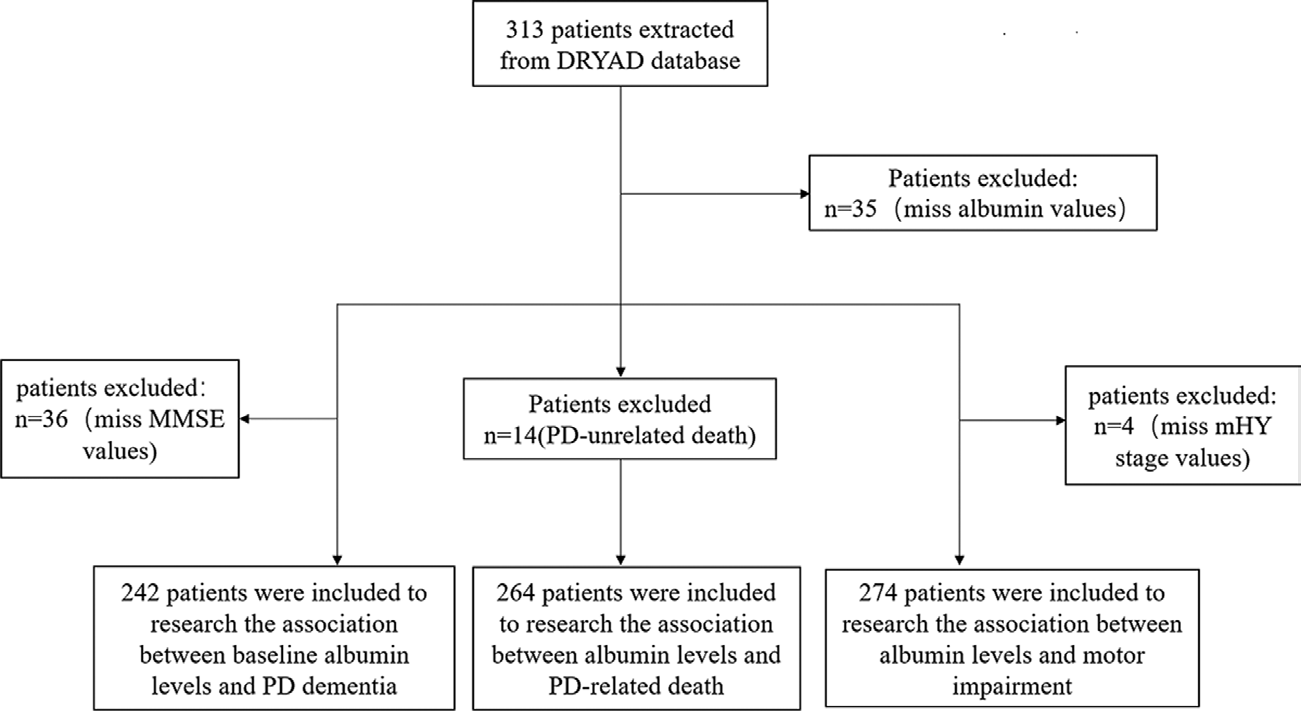
Flow diagram of the enrollment of study participants. mHY = modified Hoehn-Yahr, MMSE = Mini-Mental State Examination.

The screening and enrollment of study patients are shown in Figure 1, the baseline characteristics of participants in the secondary analysis are shown in Table 1. In the group of patients with the highest serum albumin tertile, age was significantly lower than in other groups; however, the percentage of patients with 1 to 3 mHY stage and those who died from PD-related causes in the highest serum albumin tertile group were significantly higher than other groups. CRP levels and MMSE scores were significantly lower in the group with the lowest serum albumin tertile than other 2 tertiles group. The sex ratio, PD duration, and use of NSAIDs were not significantly different among the groups of albumin tertiles.

**Table 1:** Baseline characteristics of participants in the study of PD-related death by categories of serum albumin levels.

Variables	Total participants	Serum albumin tertiles (mg/dL)			*P* value
		Q1 (2.8–3.8)	Q2 (3.9–4.1)	Q3 (4.2–5.1)	
Participants (n)	264	87	69	108	
Age (years)	68.7 ± 9.4	70.3 ± 9.4	69.8 ± 9.5	66.7 ± 8.9	.015*
Sex, n (%)					.985
Female	155 (58.7)	51 (58.6)	40 (58)	64 (59.3)	
Male	109 (41.3)	36 (41.4)	29 (42)	44 (40.7)	
Duration (years)	7.0 (3.8, 10.0)	7.0 (4.0, 10.0)	8.0 (5.0, 10.0)	6.0 (3.0, 10.2)	.183
mHY, n (%)					.004*
1–3	164 (62.1)	45 (51.7)	37 (53.6)	82 (75.9)	
4–5	96 (36.4)	40 (46)	31 (44.9)	25 (23.1)	
NSAIDs, n (%)					.949
No	221 (83.7)	72 (82.8)	57 (82.6)	92 (85.2)	
Yes	39 (14.8)	13 (14.9)	11 (15.9)	15 (13.9)	
unknown	4 (1.5)	2 (2.3)	1 (1.4)	1 (0.9)	
CRP (mg/L)	0.5 (0.2, 1.0)	0.8 (0.4, 2.2)	0.4 (0.2, 0.8)	0.4 (0.2, 0.7)	<.001*
MMSE	25.0 (23.0, 28.0)	25.0 (21.0, 27.0)	25.0 (24.0, 29.0)	25.0 (24.0, 28.0)	.006*
PD-related death					.009*
Alive	229 (86.7)	71 (81.6)	56 (81.2)	102 (94.4)	
Death	35 (13.3)	16 (18.4)	13 (18.8)	6 (5.6)	

Data are presented as mean ± SD, median (Q1–Q3), or n lrb%.

CRP = C-reactive protein, Duration = PD disease duration, mHY stage = modified Hoehn-Yahr stage, NSAIDs = nonsteroidal anti-inflammatory drugs, MMSE = Mini-Mental State Examination, outcome = PD-related death.

*P < 0.05.

### 3.2. Association of serum albumin levels with cognitive function

The Bs and 95% CIs for the level of cognitive function determined by serum albumin levels are shown in Table 2. In the nonadjusted model and adjusted I model, with the increasing concentration or tertile of serum albumin, MMSE scores was growing. In the adjusted II model that adjusted for age, sex, PD disease duration, mHY stage, use of NSAIDs, and CRP, MMSE scores of patients of the middle tertile group increased 2.09 compared to patients of the lowest tertile group [B 2.09 (95% CI 0.45,3.73), (*P* = .013)]; however, there was insignificant difference in MMSE scores between the highest tertile group and lowest tertile group.

**Table 2:** Association between baseline serum albumin levels and MMSE.

	Nonadjusted	*P* value	Adjust I	*P* value	Adjust II	*P* value
Albumin (mg/dL)	2.52 (1.02, 4.02)	.001*	1.79 (0.34, 3.24)	.017*	1.47 (−0.08, 3.01)	.064
Albumin levels tertiles						
Q1 (2.8–3.7 mg/dL)	1		1		1	
Q2 (3.8–4.1 mg/dL)	2.21 (0.55, 3.87)	.01*	2.29 (0.71, 3.86)	.005*	2.09 (0.45, 3.73)	.013*
Q3 (4.2–5.1 mg/dl)	2.61 (0.97, 4.25)	.002*	2.03 (0.46, 3.59)	.0012*	1.62 (−0.05, 3.28)	.058
*P* for trend	.004		.028		.138	

Data presented are Bs and 95% CIs; Adjust I model adjusted for age and sex; adjust II model adjusted for adjusted I + PD disease duration + modified Hoehn-Yahr stage + nonsteroidal anti-inflammatory drugs + C-reactive protein.

B = partial regression coefficient, CI = confidence interval.

*P < 0.05.

### 3.3. Association of serum albumin levels with motor impairment

Smoothing curves of serum albumin levels against severe motor impairment (mHY stages ≥ 4) were plotted, after adjusting for sex, age, PD disease duration, MMSE scores, use of NSAIDs, and CRP, continuous linear association was observed (Figure 1A, Supplemental Digital Content, http://links.lww.com/MD/H148). Table 3 presented the ORs and 95% CIs for the risk of severe motor impairment determined by serum albumin levels. In the nonadjusted model, adjusted I model and II model, as concentration of serum albumin increased, the risk of severe motor impairment was decreased. The risk of severe motor impairment between the middle tertile group and lowest tertile group was not significantly different in all 3 models (Table 3). In group of patients with the highest albumin tertile, the risk of severe motor impairment is lower than those in the lowest tertile group after adjustment for age, sex, PD disease duration, MMSE, use of NSAIDs and CRP [OR 0.37 (95% CI 0.17, 0.78) (*P* = .009)].

**Table 3:** Association between baseline serum albumin levels and severe motor impairment.

	Nonadjusted	*P* value	Adjust I	*P* value	Adjust II	*P* value
Albumin (mg/dL)	0.26 (0.14–0.51)	<.001*	0.32 (0.16–0.63)	.001*	0.34 (0.14–0.8)	.013
Albumin levels tertiles						
Q1 (2.8–3.8 mg/dL)	1		1		1	
Q2 (3.9–4.1 mg/dL)	0.93 (0.5–1.74)	.826	0.96 (0.5–1.83)	.896	0.64 (0.28–1.44)	.278
Q3 (4.2–5.1 mg/dL)	0.36 (0.2–0.64)	.001*	0.41 (0.22–0.76)	.004*	0.37 (0.17–0.78)	.009*
*P* for trend	.001*		.004*		.009*	

Data presented are ORs and 95% CIs; Adjust I model adjusted for age and sex; adjust II model adjusted for adjusted I + PD disease duration + MMSE scores + nonsteroidal anti-inflammatory drugs + C-reactive protein.

CI = confidence interval, MMSE = Mini-Mental State Examination, OR = odds ratio, PD = Parkinson disease.

*P < 0.05.

### 3.4. Association of serum albumin levels with PD-related death

**Figure 2.**
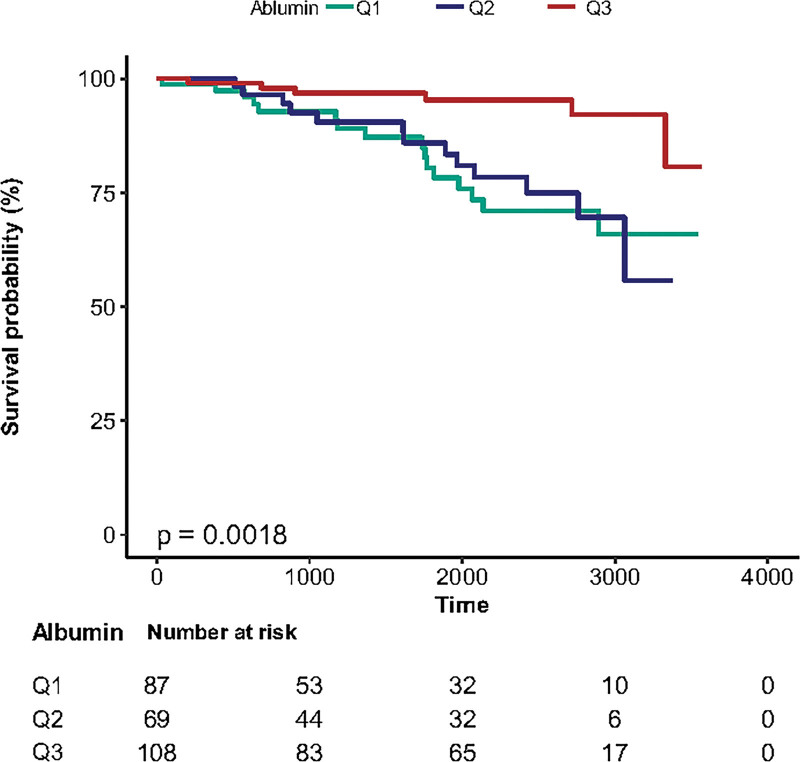
Survival curves (PD-related death) of patients with PD based on baseline albumin tertiles. Note: Q1 = (2.8–3.8 mg/dL); Q2 (3.9–4.1 mg/dL); Q3 (4.2–5.1 mg/dL).

The association of serum albumin levels with PD-related death is shown in Table 1, Supplemental Digital Content, http://links.lww.com/MD/H149, and the survival curves of patients with PD based on baseline albumin tertiles are shown in Figure 2. In the nonadjusted model, adjusted model I, and adjusted model II, the risk of incident PD-related death increased in the highest tertile group of serum albumin compared to the lowest tertile group, and the HRs were [0.23 (0.09, 0.58) *P* = .002], [0.22 (0.09, 0.57) *P* = .002], and [0.3 (0.1, 0.83) *P* = .021], respectively (Table 1, Supplemental Digital Content, http://links.lww.com/MD/H149). The Kaplan–Meier survival curves for tertiles of albumin concentration showed significantly higher survival rates for patients with the highest tertile of albumin compared with those with the lower tertiles (*P* = .0018, Fig. 2).

### 3.5. Threshold effect analysis of serum albumin levels on cognitive function

To clarify the dose–response relationship between serum albumin and cognitive function, a smoothing function analysis was performed. A nonlinear relationship between serum albumin levels and MMSE scores was observed after adjusting for potential confounding factors (Figure 1B, Supplemental Digital Content), http://links.lww.com/MD/H148).

MMSE was positively correlated with the concentration of serum albumin when it was <3.9 mg/dL [B 6.823 (1.654, 11.692) *P* = .007]. However, when the serum albumin level was >3.9 mg/dL, the B for MMSE was −1.725 (−4.756, 1.306, *P* = .263), indicating the absence of a statistically significant association between MMSE and albumin level (Table 4). In Figure 1B, Supplemental Digital Content, http://links.lww.com/MD/H148, the solid line indicates MMSE values, and the dashed lines represent pointwise 95% CIs adjusted for age, sex, PD disease duration, modified HY stage, use of NSAIDs, and CRP.

**Table 4:** Threshold effect analysis of serum albumin levels on MMSE.

Cognitive function	B (95% CI)	*P* value
One-line logistic regression model	1.47 (−0.08, 3.01)	.064
Two-piecewise logistic regression model		
Albumin < 3.9 (mg/dL)	6.823 (1.654, 11.692)	.007*
Albumin ≥ 3.9 (mg/dL)	−1.725 (−4.756, 1.306)	.263
Likelihood ratio test		.008*
Nonlinear test		.033*

Adjusted for age, sex, PD disease duration + modified Hoehn-Yahr stage, nonsteroidal anti-inflammatory drugs, C-reactive protein.

B = partial regression coefficient, CI = confidence interval, MMSE = Mini-Mental State Examination, PD = Parkinson disease.

*P < 0.05.

### 3.6. Subgroup analyses

We performed stratified analyses and interactive analyses (Table 2, Supplemental Digital Content, http://links.lww.com/MD/H150) to determine whether the association between serum albumin levels and PD-related death was stable in different subgroups. In the association between serum albumin and incident PD-related death, an interactive role existed in any subgroup; however, ORs were lower in males, older patients (≥65 years), or patients with PDD (MMSE ≤ 24), and the highest tertile of serum albumin, specifically 0.09 (0.01, 0.75), 0.16 (0.03, 0.75), and 0.17 (0.03, 0.92), respectively. Meanwhile, in the group with the highest CRP levels (≥0.5 mg/L) and the longest PD disease duration (7–29 years), those patients with serum albumin levels in the top tertile had a lower risk of PD-related death, and the ORs were 0.21 (0.07, 0.65) and 0.3 (0.1, 0.87).

## 4. Discussion

In this secondary analysis of a previous retrospective study conducted in Japan,^[11]^ we observed a nonlinear relationship between serum albumin levels and MMSE; also, we determined the threshold value. Our results indicate that higher serum albumin levels are associated with higher MMSE scores when the albumin level is <3.9 mg/dL, and was not related to MMSE when it exceeded 3.9 mg/dL, independent of age, sex, mHY stage, PD disease duration, CRP level, and use of NSAIDs. Meanwhile, serum albumin was also related to a decreased risk of PD-related death after adjustment for age, sex, mHY stage, PD disease duration, CRP, MMSE, and use of NSAIDs, with a median follow-up of 5.24 years; also, this relationship was more significant in older (≥65 years) or male patients, as well as patients with prolonged PD disease duration (7–29 years) ad a higher CRP baseline level (≥5 mg/L) when serum albumin was between 4.2 and 5.1 mg/dL.

Our study showed an association between serum albumin levels with MMSE, mHY stages and PD-relative death of PD patients, and the potential protective role of albumin in cognitive function, motor impairment, and PD-related death. PD and AD both are common neurodegenerative disorders, the previous studies have shown the role of albumin in AD. Albumin levels were lower in the AD group than in the control group,^[13]^ and the scores of the Korean version of the MMSE showed a positive correlation with albumin level in the AD group.^[14]^ Jia-Jyun Wu et al also found that dementia severity was significantly associated with serum albumin level in the oldest-old with AD.^[6]^ Albumin levels appear to be associated with other types of dementia; indeed, hypoalbuminemia is a risk factor for dementia in patients on hemodialysis.^[15]^ Meanwhile, in older people with dementia receiving enteral nutrition, an increased albumin level was associated with decreased mortality.^[16]^ The level of serum albumin has been reported to be significantly lower in patients with PD than in healthy controls (*P* = .001), and it is an independent risk factor for PD (*P* ≤ .001).^[17]^ Albumin is also an indicator of nutritional condition, and patients with PD who were malnourished had lower albumin levels, longer course of disease, higher HY stage, and lower cognitive scores than patients with normal nutrition.^[18]^ All the aforementioned studies suggested that albumin has a potential protective role in dementia and mortality, partially supporting our results.

Previous study had shown that lower albumin levels were associated with an increased all-cause mortality risk in community-dwelling older adults.^[19]^ In our study, we also research the relationship between serum albumin levels and all-cause death in PD patients. The Kaplan–Meier survival curves of all-cause death showed significantly higher survival rates for patients with the highest tertile of albumin compared with those with the lower tertiles (Figure 2, Supplemental Digital Content, http://links.lww.com/MD/H151). In nonadjusted model, higher albumin levels were related with the lower possibilities of all-cause death, but this significant relationship was not shown by the model II which adjusted by age, sex, PD disease duration, mHY stage, use of NSAIDs, CRP, MMSE (Table 3, Supplemental Digital Content, http://links.lww.com/MD/H152), but all OR values were <1, so insignificant association between baseline albumin level and all-cause death in model II might due to the small sample size of the study.

The mechanisms underlying the pathophysiology of PD are far from fully understood; however, the protein aS is a crucial component, and it may further induce oxidative stress and neuroinflammation, which also play critical roles in the pathogenic mechanism of PD.^[20–22]^ Albumin is the most abundant plasma protein in the body and a major component of cerebrospinal fluid. Also, it has been reported that HSA could break the catalytic cycle that promotes αS self-association, and remodeling of aS oligomer and high-MW fibrils into chimeric intermediates, with reduced toxicity and suppressed membrane interactions with the N-terminal and central αS regions, thus inhibiting αSn toxicity.^[10]^ Meanwhile, HAS could impede fibrillization and mitigate membrane damage of αS,^[23]^ as well as reduce the aggregation of αS significantly at the concentration found in the human serum.^[9]^ The antioxidant and anti-inflammatory activities of HSA have been reported in the literature, and its neuroprotective role in stroke and AD has been supported by many studies.^[4,24]^ Human nonmercaptalbumin (HNA) (directly oxidized form of HSA-to-HSA ratio was significantly increased in idiopathic PD compared with that in healthy controls.^[25]^ The neuroprotection of albumin in PD has been reported in the aforementioned studies and may partially explain the results of this study; however, further research is needed to confirm this finding.

It is possible that some important covariates may not be included in our research, which is limited by the original data. However, similar to the original study, the most important parameters have been included, such as age, sex, PD duration and severity, cognitive function, and inflammatory state; thus, our results have some clinical value. To our knowledge, this is the first study to investigate the relationship between albumin and cognitive function, as well as the survival outcome of PD; however, future randomized controlled studies with large populations are needed.

## 5. Conclusions

In conclusion, an increase in the serum albumin level when it was < 3.9 mg/dL was independently associated with better cognitive function. Meanwhile, elevated serum albumin levels were independently related to a lower degree of motor impairment and incidence of PD-related death.

## Acknowledgments

The authors really appreciate the data providers of the study. They completed the entire study. They are Hideyuki Sawada*(corresponding author), Tomoko Oeda, Atsushi Umemura, Satoshi Tomita, Masayuki Kohsaka, Kwiyoung Park, Kenji Yamamoto, Hiroshi Sugiyama (the rankings of these investigator were ranked according to the original study).

## Author contributions

Conceptualization, Shujun Sun and Yiyong Wen; methodology, Shujun Sun and Yiyong Wen; software, Shujun Sun.; validation, Shujun Sun and Yandeng Li; formal analysis, Shujun Sun and Yiyong Wen; investigation, Shujun Sun and Yandeng Li; resources, Shujun Sun and Yandeng Li; writing—original draft preparation, Shujun Sun, Yiyong Wen; writing—review and editing, Shujun Sun, Yiyong Wen; visualization, Yandeng Li; supervision, Yiyong Wen; project administration, Shujun Sun; funding acquisition, Shujun Sun. All authors have read and agreed to the published version of the manuscript.

## Supplementary Material


