# Supplementary figures and images for: Serum albumin, cognitive function, motor impairment, and survival prognosis in Parkinson disease
Source: Medicine (Baltimore). 2022 Sep 16;101(37):e30324. doi: 10.1097/MD.0000000000030324 (PMC9478219; doi:10.1097/MD.0000000000030324)

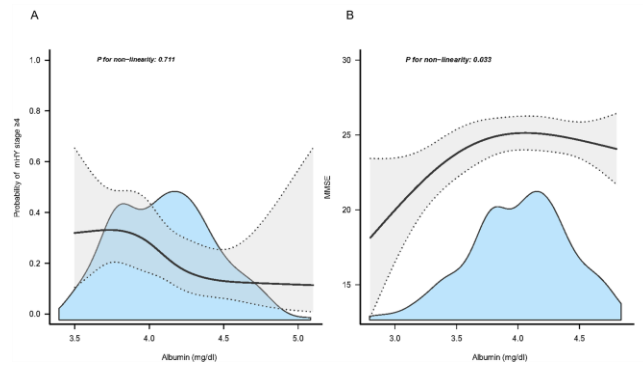

Supplement: Supplementary file 1 [file medi-101-e30324-s001.pdf]

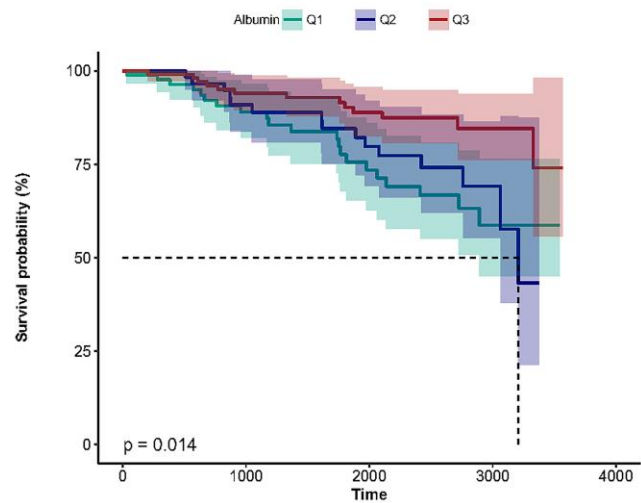

| Number at risk |     |    |    |    |   |
|----------------|-----|----|----|----|---|
| Q1             | 92  | 55 | 34 | 10 | 0 |
| Q2             | 71  | 45 | 33 | 7  | 0 |
| Q3             | 115 | 87 | 66 | 17 | 0 |

Supplement: Supplementary file 4 [file medi-101-e30324-s004.pdf]
