# Supplementary material for: Serum albumin, cognitive function, motor impairment, and survival prognosis in Parkinson disease
Source: Medicine (Baltimore). 2022 Sep 16;101(37):e30324. doi: 10.1097/MD.0000000000030324 (PMC9478219; doi:10.1097/MD.0000000000030324)
Supplement: Supplementary file 2 [file medi-101-e30324-s002.pdf]

Supplemental Table 1: Association between baseline serum albumin levels and PD-related death

	Nonadjusted	P value	Adjust I	P value	Adjust II	P value
Albumin n(mg/dl)	0.26 (0.12,0.58)	< 0.001 *	0.26 (0.12,0.58)	< 0.001 *	0.36 (0.15,0.86)	0.021 *
Albumin level tertiles						
Q1(2.8-3.8mg/dl)	1		1		1	
Q2(3.9-4.1mg/dl)	0.96 (0.46,1.99)	0.904	0.87 (0.41,1.82)	0.705	0.89 (0.37,2.14)	0.787
Q3(4.2-5.1mg/dl)	0.23 (0.09,0.58)	0.002 *	0.22 (0.09,0.57)	0.002 *	0.3 (0.1,0.83)	0.021 *
P for trend	0.002		0.001		0.022	

Notes: Data presented are HRs and 95% CIs; Adjust I model adjusted for age and sex; adjust II model adjusted for adjusted I + PD disease duration+ modified Hoehn-Yahr stage+ non-steroidal anti-inflammatory drugs+ C-reactive protein+ Mini-Mental State Examination.
