# Supplementary material for: Serum albumin, cognitive function, motor impairment, and survival prognosis in Parkinson disease
Source: Medicine (Baltimore). 2022 Sep 16;101(37):e30324. doi: 10.1097/MD.0000000000030324 (PMC9478219; doi:10.1097/MD.0000000000030324)
Supplement: Supplementary file 3 [file medi-101-e30324-s003.pdf]

Supplemental Table 2. Subgroup analyses of the association between serum albumin levels and PD-related death.

Confounding factor category	Serum albumin tertiles (mg/dl)			P for trend	P for interaction
	Q1(2.8-3.8)	Q2(3.9-4.1)	Q3(4.2-5.1)		
Age（years）					0.341
<65	1 (reference)	2.19 (0.22,22.29)	0.94 (0.17,5.18)	0.808	
≥65	1 (reference)	1.25 (0.52,3.02)	0.16 (0.03,0.75)	0.034 *	
Sex					0.157
Female	1 (reference)	1.25 (0.34,4.67)	0.47 (0.13,1.73)	0.23	
Male	1 (reference)	1.23 (0.36,4.22)	0.09 (0.01,0.75)	0.021 *	
mHY					0.732
1-3	1 (reference)	0.91 (0.19,4.33)	0.27 (0.06,1.28)	0.083	
4-5	1 (reference)	1.82 (0.61,5.42)	0.37 (0.1,1.41)	0.286	
Duration (years)					0.974
Q1(0.5-6)	1 (reference)	0.37 (0.03,4.49)	0.34 (0.02,4.64)	0.37	
Q2(7-29)	1 (reference)	1.26 (0.51,3.15)	0.3 (0.1,0.87)	0.035 *	
MMSE					0.755
≤24	1 (reference)	1.57 (0.33,7.47)	0.17 (0.03,0.92)	0.05	
＞25	1 (reference)	1.33 (0.46,3.88)	0.4 (0.11,1.52)	0.241	
CRP (mg/l)					0.367
<5	1 (reference)	3.34 (0.61,18.15)	0.93 (0.11,7.65)	0.919	
≥5	1 (reference)	0.67 (0.22,2.07)	0.21 (0.07,0.65)	0.006 *	

Notes: adjusted for age, sex, PD disease duration, modified Hoehn-Yahr stage, non-steroidal anti-inflammatory drugs, C-reactive protein. Abbreviations: Duration, PD disease duration; mHY, modified Hoehn-Yahr stage; MMSE, Mini-Mental State Examination; CRP, C-reactive protein.
