# Supplementary material for: Serum albumin, cognitive function, motor impairment, and survival prognosis in Parkinson disease
Source: Medicine (Baltimore). 2022 Sep 16;101(37):e30324. doi: 10.1097/MD.0000000000030324 (PMC9478219; doi:10.1097/MD.0000000000030324)
Supplement: Supplementary file 5 [file medi-101-e30324-s005.pdf]

Supplemental Table 3: Association between baseline serum albumin levels and all-cause death

	Non-adjusted	P value	Adjust I	P value	Adjust II	P value
Albumin n(mg/dl)	0.37(0.19~072)	0.004*	0.38(0.19,0.75)	0.006*	0.62(0.29,1.31)	0.21 *
Albumin level tertiles						
Q1(2.8-3.8mg/dl)	1		1		1	
Q2(3.9-4.1mg/dl)	0.85 (0.44~1.65)	0.631	0.76 (0.39~1.49)	0.425	0.82 (0.38~1.76)	0.607
Q3(4.2-5.1mg/dl)	0.38 (0.19~0.76)	0.006*	0.38 (0.19~0.77)	0.007*	0.61 (0.28~1.36)	0.228
P for trend	0.006		0.006		0.228	

Notes: Data presented are HRs and 95% CIs; Adjust I model adjusted for age and sex; adjust II model adjusted for adjusted I + PD disease duration+ modified Hoehn-Yahr stage+ non-steroidal anti-inflammatory drugs+ C-reactive protein+ Mini-Mental State Examination.
